# Machine Learning in Computational Design and Optimization of Disordered Nanoporous Materials

**DOI:** 10.3390/ma18030534

**Published:** 2025-01-24

**Authors:** Aleksey Vishnyakov

**Affiliations:** 1 Aramco Innovations LLC, 119234 Moscow, Russia; avishnja@polly.phys.msu.ru; 2 Department of Physics, Moscow State University, 119134 Moscow, Russia

**Keywords:** porous materials, active carbons, mesoporous oxides, aerogels, microporous polymers, gas separation, machine learning

## Abstract

This review analyzes the current practices in the data-driven characterization, design and optimization of disordered nanoporous materials with pore sizes ranging from angstroms (active carbon and polymer membranes for gas separation) to tens of nm (aerogels). While the machine learning (ML)-based prediction and screening of crystalline, ordered porous materials are conducted frequently, materials with disordered porosity receive much less attention, although ML is expected to excel in the field, which is rich with ill-posed problems, non-linear correlations and a large volume of experimental results. For micro- and mesoporous solids (active carbons, mesoporous silica, aerogels, etc.), the obstacles are mostly related to the navigation of the available data with transferrable and easily interpreted features. The majority of published efforts are based on the experimental data obtained in the same work, and the datasets are often very small. Even with limited data, machine learning helps discover non-evident correlations and serves in material design and production optimization. The development of comprehensive databases for micro- and mesoporous materials with low-level structural and sorption characteristics, as well as automated synthesis/characterization protocols, is seen as the direction of efforts for the immediate future. This paper is written in a language readable by a chemist unfamiliar with the data science specifics.

## 1. Introduction

Nanoporous materials find multifarious applications in separations, catalysis, gas and liquid storage, insulation, the semiconductor industry, energy storage, and so on. Their pore sizes range from angstroms to 100 nm, and their porosity may reach 99%+ [1]. The body of published results related to the synthesis and characterization of porous materials is really vast, and the methods are reasonably standardized and well developed, which prompts the employment of this knowledge for the data-driven design of materials tailored for specific purposes. The stochastic optimization of potential zeolite structures by Eral and Deem [2] is a pioneering work in the data-driven design of porous adsorbents: the authors aimed at predicting new (“potential”) zeolite structures based on the structures of known ones using Monte Carlo (MC) optimization. Although the machine learning (ML) term was not mentioned, the optimization did not involve any calculations of specific chemical interactions. The only target was the structural similarity of the elements of the suggested structures to those of existing zeolites. That is, the new zeolite frameworks were constructed using the structural features learned from the knowledge database. The optimization net caught fishes of different kinds: despite a multitude of chemically unstable structures, existing frameworks that were not in the training database were also recovered, and some of the suggested structures were synthesized later.

The approach was based on the crystalline nature of the targeted adsorbent, from which the structural features could be readily constructed by expert chemists. The discovery of metal–organic frameworks (MOFs [3]), covalent networks (COFs [4]), and regular porous polymer networks (PPNs [5]) created a (relatively) vast pool of structures that could be readily classified into categories, interpreted in terms of tangible descriptors, unambiguously characterized, and compiled in databases, with structural characteristics and physico-chemical properties listed, including adsorption. The lion’s share of all applications of data-driven methods to porous materials target these groups of crystalline solids. And even “overcoming data scarcity” is addressed, mostly in areas where data are comparatively abundant or straightforward to generate [6]. The crystal structures of synthesized compounds have been collected in several databases, general [7,8,9,10] or material-specific [11,12,13]. These studies have been described in numerous recent reviews [14,15,16,17,18,19] and are not the target of this paper.

At the same time, a substantial effort has already been invested into the ML-assisted design, characterization and optimization of porous materials that are disordered in at least one aspect important for their function. That is, a disordered mesoporous silica with walls built from a zeolite [20] has ordered microporosity but disordered mesoporosity (here, we apply “micro-, meso- and macroscale” as commonly accepted in adsorption science, with micropores ranging from angstroms to 2 nm, mesoscale porosity below 100 nm, and macroscale is everything larger than that [21]). Disordered porous solids form numerous dissimilar groups, and despite an enormous body of experimental and modeling results on their structure and properties, the results are not easily interpreted with clear descriptors. The chemical space of microporous crystals and templated ordered mesoporous materials [22] is discrete. The space of disordered porous materials is continuous, and so is the space of their chemical and physical properties. All descriptions of disordered porous materials that the author is aware of are ill posed. Many different structures can be proposed that have the same (within experimental/modeling errors) porosity, density, specific surface area (SSA), X-ray diffractogram (XRD), acoustic properties, etc. At present, these navigation problems are making disordered materials not very attractive to data scientists despite their practical importance. For example, aerogels have way more industrially important applications than all MOFs, COFs, and PPNs combined.

Goals for the data-driven design and optimization of irregular porous materials: The focus of this review is the computational design and optimization of the synthesis and characterization of disordered porous materials. Designed are, obviously, the pore structure and the properties of the solid phase that comprises the structure. The goal is finding an available structure that provides for the desired properties. Optimization rather applies to the initial reactant formulation or to the conditions of synthesis needed in order to achieve the desired structure and therefore the desired properties as closely as possible. Therefore, most efforts reviewed have aimed at either obtaining a particular material or, sometimes, the optimization of the manner of production, although direct industrial applications of ML to heterogeneous porous solids are still to be found.

**Figure 1:**
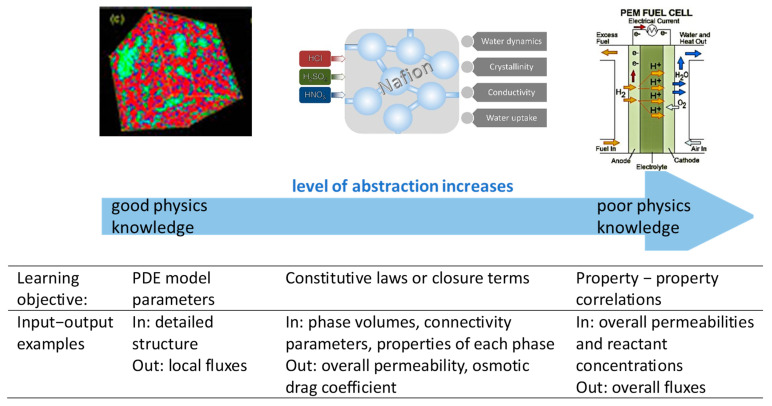
Examples of different approaches to ML of fluid transport in heterogeneous porous media depending on the knowledge of the medium structure and understanding of the physics of the process, here shown on the example of “dynamically nanoporous” [29,30]. Nafion membrane with the nanoporous structure formed via nanoscale segregation of hydrated polymer. Left: most detailed approach with the nanopore structure available from structural simulations. Middle: volumes of the mobile and immobile subphases and some connectivity characteristics are available. Right: entirely homogenized model characterized with macroscopic dynamic properties. As the spatial and temporal scales increase, and the detailed structure of the porous matrix is unavailable or too complex to account for, non-interpretable entirely data-based model linking the input pressures and concentrations to the fluxes are applied. Drawn according to ref. [28]. Images from refs: [31] (ACS permission) [32]; (CCL); [33] (CCL).

The main directions of the efforts outlined in the literature: There are several general reviews on the data-driven discovery and optimization of porous materials that also mention disordered solids. Beyond general expectations (e.g., [23,24]) and interesting methodological developments [25,26], a recent collection [27] gives a broad overview of the main pathways to apply data-based techniques (stand-alone or in conjunction with physics-driven modeling) to heterogeneous porous materials. In particular, the following directions of effort are suggested as potential breakthrough areas for the next ten years (out of these ten, three years have already passed, and for data science, three years is a long time):

(A) Discovering the governing equations with ML [28] (Figure 1). Active ML can “interrogate” complex processes, which is “particularly useful for the analysis of highly heterogeneous, anisotropic materials, where idealized descriptions often fail”. Multi-dimensional, ill-posed problems are where machine learning often excels. Dealing with the uncertainty of the material’s structure remains a major challenge that seems unsurmountable at present: one must deal with many different realizations of the same process using different possible material structures that fall within the same description, and somehow ensure that the structure chosen for modeling is representative (computational costs are disregarded for the moment). The alternative is a physics-based homogenized model, which devalues the entire data-driven approach. Positive examples mentioned by D’Elia [28] do not involve heterogeneous porous materials. Unfortunately, in the current review, we have not found any papers that seem promising in terms of discovering the governing equations.
(B) Data-driven acceleration of simulations in complex, multi-scale porous media [34] is certainly a promising and already fruitful direction. The main goal of ML is to optimize material structure/chemistry, etc. Due to difficulties in generalizing simulation results due to data navigation challenges, we need fast and computationally efficient tools to simulate processes of interest in various structural realizations. Surrogate data-based models outperform traditional ODE/PDE solvers in many cases, from fluid flow [35] to chemical kinetics [36]. Multi-physics phenomena involving drastically different spatial and temporal scales are especially problematic. At smaller scales, ML can reasonably replace physics-based modeling. Furthermore, the rapid development of physics-informed ML (PIML) [37] allows for the integration of physics and data-driven approaches. PIML is expanding to new types of systems and media, and its scope is already wide enough to allow for the design and optimization of disordered porous materials.
(C) Machine learning structure–property relationships [38]. This field has already been well established. Numerous studies have focused on predicting the transport and mechanical properties of disordered porous materials using microscopic images. However, current practices show that these predictions are usually specific to a particular material, and the results obtained for one group of materials can’t be generalized to other groups, making it challenging to develop a more general approach. Physics-based modeling, such as Lattice-Boltzmann (LB) simulations for permeability [39] and finite element analyses (FEM) for elastic moduli, often serve as ground truth for these predictions. Nevertheless, obtaining representative samples for learning databases remains challenging. For example, rock physics data models are often based on a small number of samples that are cut and shifted using a sliding window technique to increase the training set size.

There are other challenges unnoticed in the previous reviews:

(D) ML of property–property relationships. That is, the properties that can be easily measured are related to those that are difficult to determine experimentally or costly to model. For instance, the reconstruction of physical fields from real-time observations at a few locations has a long history (a brief review can be found in ref. [40]). Additionally, PIML enables the relationship between different physical fields to be established. This review contains a few useful examples.
(E) Linking the initial formulations and synthesis condition to structural parameters of the resulting materials. The review below demonstrates that this is the most active area of application of ML techniques to disordered nanoporous materials at present (as expected, given that such relationships are complex, nonlinear, and almost impossible to model based on chemistry/physics alone).

Directions (A) and (B) explicitly rely on a thorough understanding of the structural properties of the material and the physical processes involved. Directions (C) may use physical models or be based entirely on data. Property–property relationships (Challenge D) are more difficult to establish compared to structure–property relationships, but properties can be understood through physical models of corresponding processes for dimensionality reduction. Materials synthesis (Challenge E) is commonly too complex, and data-based models summarize the available empirical knowledge.

This article does not target giving a “bold broad view” on the perspectives of ML application in the computational design of heterogeneous porous materials, but rather analyzes the current practices and compares them to the previous expectations. It more focuses on materials synthesis than on porous media in general (recent review [41] or specific applications (e.g., ref. [42,43,44,45,46]). It aims to review:

(i) general approaches: what exactly is targeted currently and why (research driven by practical needs *vs.* research driven by methodological interest)
(ii) material types
(iii) data sources
(iv) ML methods and optimization techniques applied in the studies of interest
(v) achievements and problems

To the best or our knowledge, the efforts tackling challenges (D,E) in disordered porous materials have never been reviewed. Special attention is paid to the relations between the data-based models and physic-based models as described in Figure 1.

The paper is structured as follows: Section 2 gives an overview of ML application in nanoporous materials characterization; Section 3 is devoted to active carbons, Section 4 describes microporous polymers used in gas separation, Section 5 considered mesoporous oxides, aerogels, etc. Section 6 summarizes the review; Appendix A is a table that summarizes the current literature; Appendix B contains a glossary of ML methods applied in materials characterization, design and optimization, tailored for a chemist unfamiliar with data science.

## 2. ML in Characterization of Disordered Porous Materials

Facile and inexpensive analysis of the resulting porosity is essential for optimizing the synthesis of porous materials. ML based techniques are mainly used to reconstruct a 3D digital map of the porous matrix, primarily in binary and multiclass segmentation. Computer tomography (CT) as an inverse problem was reviewed quite recently [47] and generally is not specific to nanoscale, as well as the techniques for the evaluation of material macroscopic characteristics from images, which will be considered elsewhere. The most common method for 3D reconstruction of nanoporous materials is focused ion beam (FIB)-scanning electron microscopy (SEM). FIB destroys the material layer by layer and SEM provides a series of consecutive cross-sectional images of the 3D sample. However, the segmentation of these cross-sectional images is complicated by the “shine-through” effect, where deeper layers affect the image. Traditional thresholding methods [48] often lead to unsatisfactory results.

In supervised machine learning, the algorithm is trained “by example”. Fager et al. [49] in their FIB-SEM characterization of ethyl cellulose/hydroxypropyl cellulose films with submicron pores, generated 3000 × 2000 pixel images. The authors manually segmented 200 256 × 256 pixel fragments of consecutive layers. Since the segmentation depends on the top layer and several neighboring layers, the authors used five neighboring images as input for the segmentation of the current top layer. To take into account the surroundings of each point, each image was convolved with Gaussian filters with standard deviations varying from 1 to 128. Together with the raw top image, this resulted in 89 features. A random forest (RF) decision tree ensemble (DTE) technique was employed to learn from the manually segmented images and segment the other layers, thereby enabling pore reconstruction. The ML approach addresses the issues of subsurface and grayscale intensity overlap in the image segmentation for 3D reconstruction. Čalkovský et al. [50] explored the significance of image contrast in ML-based FIB-SEM image segmentation, also applied to nanoporous polymer structures. The study demonstrated the superior performance of ML-driven techniques compared to traditional segmentation algorithms.

**Figure 2:**
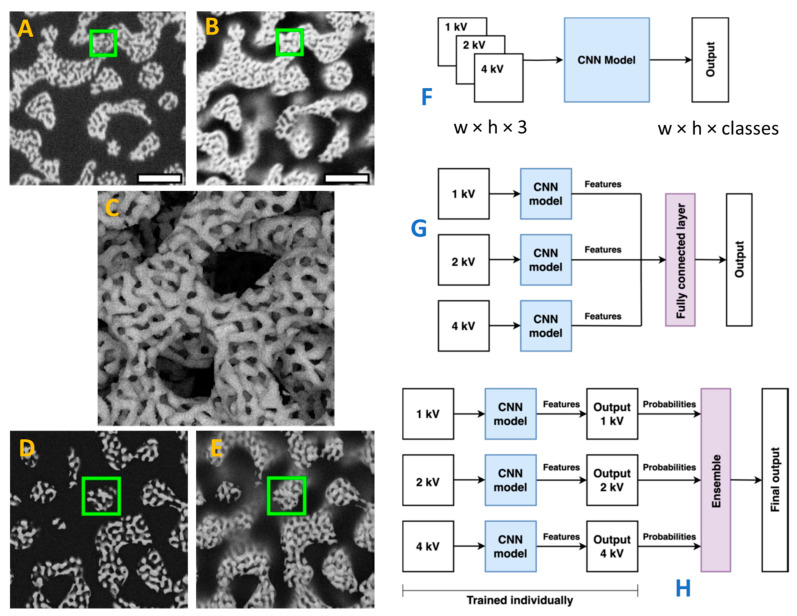
Illustration of segmentation of FIB-SEM images of HNPG using a synthetic dataset. (**A**) a example of SEM image of HNPG slice at accelerating voltage of 1 kV, bar length is 300 nm, green square is a sliding window; (**B**) Same at 4 kV (more detailed representation with a more pronounced shine-through); (**C**) simulated 3D multiscale porous structure; (**D**) simulated SEM image at the accelerating voltage of 1 kV; (**E**) Same at 4 kV; (**F**–**H**) Different multimodal ML architectures: early, intermediate, late fusion, correspondingly. Сompiled from refs. [51,52] (CCL).

A very challenging material, hierarchical nanoporous gold (HNPG), was studied by Sardhara et al. [51,52]. HNPG forms a disordered porous structure at the submicron scale, but the gold filaments themselves are porous on the 10+ nm scale. Manual segmentation of this material is time-consuming, as a large number of images must be segmented in order to collect a sufficient dataset for such a complex structure. The authors (1) obtained SEM images at different accelerating voltages (Figure 2), (2) created *in silico* porous structures similar to HNPG, and performed MC simulations of SEM on the synthetic structures. For the simulated structures, the ground truth is known, and an ML model was trained on the synthetic dataset. The simulation was based on solid principles: it was shown theoretically [53] that the microstructure can be described by a superposition of several wave vectors with the same wavelength but different random orientations. The principles of SEM are well-established and simulators are widely available. Noise and blur were added to enhance the resemblance between the simulation results and actual SEM images. Convolutional neural networks (CNNs) were trained using sliding-window techniques applied in two or three dimensions. Patches of 64 × 64 pixels were extracted and used as the final training set for the CNN. The number of neighboring slices taken into account ranged from three to nine. The CNN outperformed the traditional Otsu segmentation algorithm [54]. The authors concluded that the ML model successfully addressed the shine-through issue. Later, the same group optimized the number of layers required [55] for accurate reconstruction and analyzed the role of thickness quantification in the reconstruction of hierarchical nanoporous materials using FIB-SEM [56].

The applications of ML in characterization are not limited to images, and can be applied to various ill-posed inverse problems, such as pore characteristic reconstruction [57]. For example, Pietrow et al [58] suggested an ANN-based method for pore size distribution (PSD) calculation from positron lifetime distributions, which is a common technique for PSD. Good agreement between ML-based and traditional methods was obtained. 

Overall, ML-based methods have the potential to solve a wide range of inverse problems in the interpretation of various types of spectroscopy data. However, their application to irregular nanoporous materials is currently limited to image processing. Reliable understanding of the formation of the pore structure allows overcoming the data scarcity problem via usage of synthetic datasets.

## 3. ML in Design, Optimization, and Screening of Active Carbons

Activated carbon is a microporous material that has numerous applications in gas separation, storage, medicine and environmental protection. In the petroleum industry, carbons are important for water treatment and gasoline vapor recovery [59]. Active carbons are usually derived from organic waste products such as coconut husks and wood chips. Through pyrolysis, organic material is converted into charcoal, which is then activated to create a highly porous adsorbent. It is assumed that the active carbon structure resembles crumpled paper sheets or semi-random stacks of thin graphite plates, as shown in high-resolution images [60] and simulations [61]. The carbons typically have a high available surface area and are characterized by their PSD (extracted from adsorption isotherms [62]), crystallinity, and impurities in the form of metals and other elements. Mesoporous carbons are also available. They are produced by using mesoporous silica as a template: the pores are filled with organic liquid, which is them polymerized and subject to pyrolysis, after which the silicious matrix is dissolved [63]. A vast amount of experimental data has been generated on carbons, including information about their original organic material composition, final product composition, X-ray diffractograms (XRD), adsorption isotherms, and breakthrough curves, etc. Although attempts to utilize this wealth of knowledge are still in their early stages, progress is being made. A specific overview of ML applications for carbons in water treatment can be found in ref. [41]. Removal of organic pollutants using carbons is discussed in ref. [45], while ML screening procedures for water purification are covered in ref. [46]. A very recent study on this topic was reported in ref. [64]. Here, we focus on other applications, but we also pay attention to some important methodological papers mentioned in previous reviews. 

Because the pathways of active carbon synthesis are fairly standard, but involve many parameters, the relationship between synthesis and properties (challenge (E)) is a natural target for ML models. Here, ML helps in systematically choosing the raw materials and synthesis pathways for a particular application, involving maximum abstraction through entirely data-driven approaches (right column, Figure 1). Wang et al. [65] related the chemical composition of the source biomass, production operating parameters (organic source/activation agent mass ratio, carbonization time, carbonization temperature, activation time, and activation temperature), to the resulting characteristics of the carbons: SSA (estimated with the Brunauer-Emmett-Teller (BET) model [66] for multilayer adsorption on a *nonporous surface*), and yield. They created a database of more than 1500 different carbons using both chemical activation (baking with H_3_PO_4_, KOH, and ZnCl_2_ at 500–700 degrees Celsius) and gas activation (CO_2_ + steam). DTEs were employed to establish useful correlations. The hydrogen content in the original biomass was found to make a significant impact on the yield of gas-activated carbon, and the mass ratio has a strong influence on the SSA for chemically activated carbons. Unfortunately, the use of BET to estimate SSA is not a good choice for microporous materials [67].

Very recently, Chang and Lee [68] collected a set of 108 chemically activated carbons from their own studies and linked the synthesis procedure to the iodine adsorption number (IAN), one of the commonly accepted characteristics of granular carbons [69]. The activating agent, agent to biochar mass ratio, activating temperature and time were all treated as categorical features. Different ML regressors (artificial neural networks ANN, support vector machines SVM, DTEs) were applied in building the correlations, ANN showed the best performance. Using all features as categorical improved the precision score but obviously hindered the interpretation: all qualitative recommendations on synthesis procedures to improve IAN were not actually ML-based. In a somewhat similar effort, Lai et al. [70] collected a set of 46 carbons from the literature and related the yield and the SSA. While linking the biomass characteristics and the synthesis procedure to the results is important, a model with seven (which were not the same for the yield and SSA predictions) features to describe 46 samples can hardly be considered as predictive. Another similar effort with straw-derived carbons was described in ref. [71].

Li et al. [72] studied the relationship between the biomass characteristic and the synthesis conditions on one size and the characteristics (SSA, pore volume, micropore volume, nitrogen content) of nitrogen-doped carbons on the other side. Interestingly, the biomass source and composition turned out to be much less important than the activation chemicals and conditions. DTE based ML model performed very well. Wang et al. [73] reported ML-assisted screening of oxygen-rich porous carbons for aqueous supercapacitors. The carbons were derived from synthetic polymers, rather than from the biomass. Another attempt to relate the synthesis parameters to capacitance was reported by Yang et al. [74].

**Figure 3:**
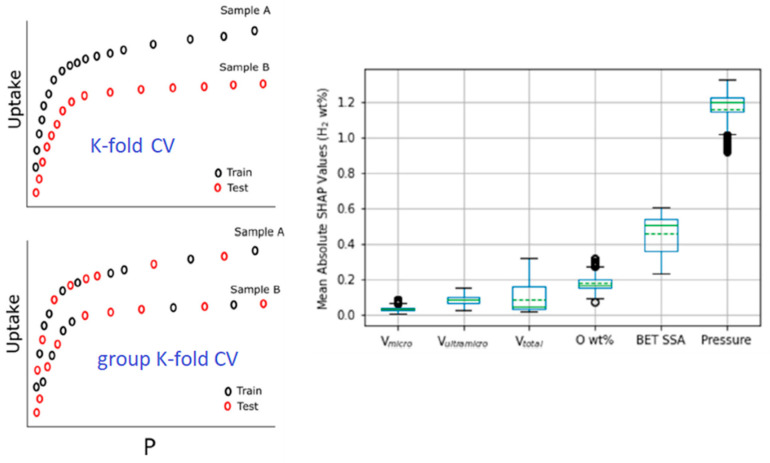
Prediction of hydrogen uptake by active carbons [77]. Left: different approaches to division of the data into training and test datasets (random selection of points *vs*. random selection of isotherms described as different approaches to stratification). The bottom approach, referred to as “group K-fold CV “, in fact implies using points of the same isotherm to predict the points from the control set. While this technique appears more precise, it is hardly relevant to the practical goal of predicting H_2_ adsorption on a new carbon sample. Right: Boxplot of feature importance values from 500 different RF models trained on different bootstrapped samples. The dashed lines show the average importance across all models, full lines show the medians, the circles show the outliers. Note that gas pressure is a feature in the model, which should lead to a noise in the predicted isotherms far exceeding the experimental error. A similar problem exists in treating the time in DTE models for chemical kinetics (see supporting info to ref. [36]). Alternatively, one may try approximation of H_2_ isotherms with adsorption models and use parameters as features. The outcome (importance of the surface area) is of course trivial from the physical point of view. Unfortunately, the predicted isotherms are not provided in the paper or supplementary materials. Compiled from ref. [77] (CCL).

ML-assisted predictions of structure–property and property–property relationships for carbons has also attracted recent attention [75,76]. For example, Kusdhany and Lyth [77] used ANN to explore hydrogen uptake in microporous carbons. They composed a set of 1700+ data points for 68 samples at 77 K and different pressures. A problem in the approach is mixing up the parameters explicitly controlled (e.g., composition) with dependents (such as the BET SSA) in the feature set and including hydrogen pressure in the feature space (Figure 3). 

Davoodi et al. [78] collected 2072 data points for H_2_ sorption by 68 active carbons. The authors paid specific attention to the element composition and ultramicropore presence. Unfortunately, the methodology shares some of the problems of ref. [77] and concluded that the pressure was the most important feature that influences hydrogen adsorption. Similar surrogate models were reported for other sorbates [43,79,80,81].

Kowalczyk et al. [82] predicted adsorption of paracetamol by active carbons from the pore size distributions using density functional theory (DFT), MC simulations and ML. The authors modelled paracetamol adsorption in carbon pores of different sizes with MC simulations, which allowed them to calculate the paracetamol sorption isotherms in any carbon with known PSD. Then the authors analyze the PSDs of actual carbons and select total SSA and the SSA formed by wide micropores and mesopores as the features that allowed a reasonable prediction of paracetamol sorption. Nanoporous carbon beads with a high surface area of supermicropores (997 m^2^/g) and mesopores (628 m^2^/g) had the highest adsorption capacity for paracetamol. While the ML itself in that paper hardly reveals anything that could not have been foretold, the work is methodically important, because it is physics-based and interpretable. The authors analyze complete adsorption isotherms, take into account the multimodality of PSDs and directly relate the performance to the adsorption mechanisms explored by MC simulations. An interpretable approach can be easily extended and is much more likely to guide synthetic effort comparable to a black box ML model fitted to a database of inconsistent characteristics.

CO_2_ sorption capacity of carbons derived from rice husk was analyzed by Palle et al [83]. Although CO_2_ at ambient conditions primarily adsorbs in micropores [84], the authors needed micropore volume, mesopore volume and SSA to fit the experimental data on CO_2_ adsorption.

Mashhadimoslem et al. [85] constructed a data-based model of sorption of N_2_, O_2_, and N_2_O on activated carbons and carbon molecular sieves. Instead of the data adsorption itself, the authors chose the parameters of the Langmuir adsorption model with two types of adsorption sites as the learning target. The main advantage of this approach is that adsorption properties of a sample are predicted from properties of other samples: points for the same sample are not a part of the training database. It also reduces the dimensionality of the problem, which is important with smaller datasets. There was a price to pay for that: the predictive ability is restricted to the Langmuir adsorption regime. The dataset in the paper in question [85] was not sufficient for significant outcomes.

**Figure 4:**
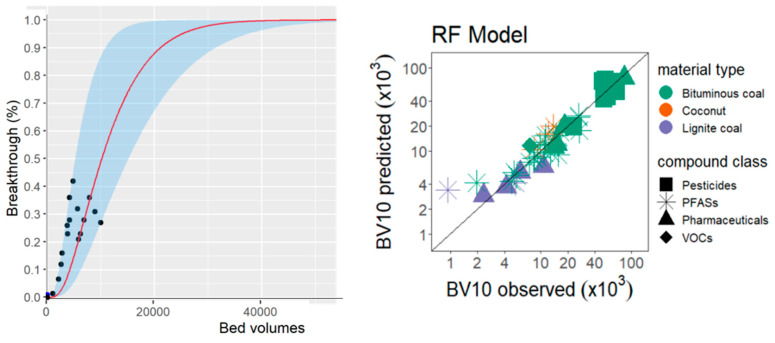
ML of pollutant permeation through granulated activated carbon bed. Left: an example of a breakthrough curve taken from the literature: black points are the raw data scanned from an original paper, red curve is the approximation with Pore-Surface Diffusion Model (PSDM [88]); light blue envelope represents the range of anticipated uncertainty of the PSDM output. Note that PSDM parameters were used in the DTE fit rather than just the raw data; bed volume is the volume of liquid solution passed through the bed. Right: test set prediction accuracy with the Random Forest approach for different carbon types and pollutant class; BV10 is bed volume of water that can be treated until MP breakthrough reached 10% of the influent MP concentration (compiled from the figures of ref. [86] and graphically edited for clarity; fair use of thesis material [86] and permission from ACS [87]).

Personal protection from various poisonous compounds and pollutants *vs.* historically one of the first applications of activated carbons. Koyama et al. [86,87] in a well-designed study, collected from the literature around 400 breakthrough curves for various pollutants on eighteen different granular organic carbon materials. Using DTEs, the authors successfully predicted early breakthrough times and related them to easily measurable properties of granulated carbons, such as the air-hexadecane partition coefficient, hydrogen bond acidity, and the dissolved organic carbon concentration in the influent water. In total, seventeen different properties were analyzed. Although the authors referred to the properties that correlated most with the breakthrough time as “top drivers”, the work is actually focused on the predictions of property—property relationships (Figure 4).

Zhou et al. [89] examined the correlation between carbon pore structure and electrical double-layer specific capacitance. They collected 70 active carbon samples from the literature. Micropore and mesopore SSA were used as features, and capacitance was the output. The scanning rate varied from 1 mV/s to 100 mV/s, with the capacitance predicted separately for each rate. At a rate of 1 mV/s, total SSA was the main factor determining capacitance, while at a rate of 100 mV/s, only mesopore area was significant.

Overall, although the results of ML applications to the structure and properties of carbon materials are still limited, there are promising trends: databases are being developed and a significant number of ML applications have clear objectives. Although ML optimization of active carbon synthesis has not led to any major breakthroughs, the statistical analysis of the role of original raw materials and synthesis processes provides a solid basis for material selection and screening, particularly for non-experts in carbon synthesis and analysis.

The published studies emphasize the importance of understanding the physical principles behind the problem and low significance of the choice of the ML techniques and the precision scores achieved. A number of papers have collected adsorption measurements for specific system types and built surrogate models to predict sorption capacity. By using points from the same isotherm for both training and testing, the precision scores are increased, creating the illusion of a predictive ability. However, any ML model within this framework is actually inferior to a spline interpolation of the isotherm, and has little to do with the actual challenges of predicting active carbon properties.

Several papers demonstrate, that reducing the “level of abstraction” (Figure 1) and employing semiempirical physics-based models as intermediates is an efficient way of dealing with data scarcity. As the problem dimensionality reduces, generalization ability increases. Potential problems are (1) making sure that the model does describe the underlying physical mechanism (2) making sure that the model does not merely hide the low precision of the measurements. For example, in ref. [86], a number of theoretical breakthrough curves were fitted to extremely scattered data. Accounting for the uncertainty of the such data in the models is a separate challenge. 

Finally, the inclusion of a substantial volumes of raw data into databases is essential for further progress. XRD data and N_2_ adsorption isotherms at 77.4 K are measured practically for every new carbon synthesized. Scientifically sound and consistent characterization of the materials a key to fruitful ML of structure–property relationship and hopefully will facilitate discovery of new relationships. PSD published in the literature are obtained with different methods and often incomparable. Obtaining consistent and interpretable pore sizes is not very straightforward even with MC and DFT kernels [90,91]. The review shows, that despite IUPAC recommendations [92], the characterization seriously limits the applicability of data-based approaches to active carbons.

## 4. Molecular Design of Microporous Glassy Polymers for Gas Separation Membranes Using Generative Neural Networks

Microporous polymers present another type of irregular porous solids. Strictly speaking, microporosity is generally found in glassy polymers [93], but the volume fraction of micropores is low and they are isolated. Polymers with long and rigid segments have, due to packing difficulties, a higher and often continuous microporosity that makes them permeable to gases. Their main applications are gas separation: due to the specifics of the structure, the microporous polymers have different permeability to different gases [94,95]. The selectivity towards gas A *vs.* gas B is the permeability ratio, which is not the same as the sorption selectivity but, very roughly, the product thereof and the diffusion coefficient ratio. The trade-off between the membrane selectivity and permeability is known as the Robeson upper bound [96] for a given gas pair. The ideal membrane polymer is one with as many pathways passable by molecular A but impassable to molecule B as possible, which can be interpreted in terms of PSD and pore throat distributions. 

Because of the practical importance, in particular to gas industry gasping for efficient CO_2_/CH_4_+C2H_6_/H_2_S separators, microporous polymer membranes are well studied, and a substantial experimental learning base is available. Unlike carbons, the MPs are determined by their chemical formula. This radically change the approaches to *in silico* design, which can be carried out as a search for the best monomer in the chemical space, somewhat similarly to the modern methods of computational drug design [97]. Still, due to very slow relaxation [98] of the polymer chains, the properties of the material depend also on manufacture process rather than on the backbone chemistry only. 

**Figure 5:**
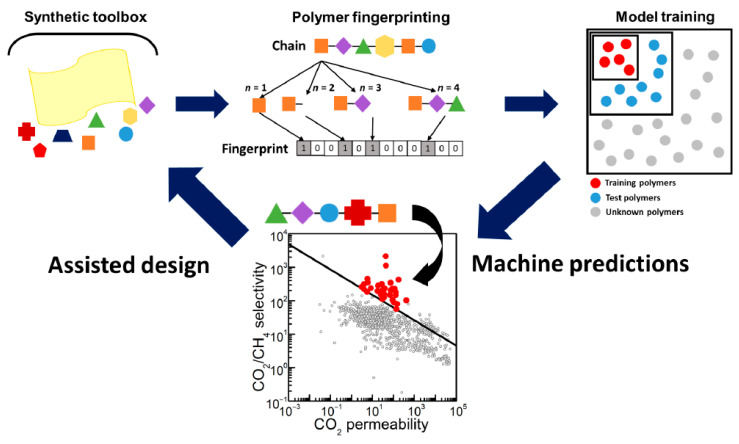
ML-assisted design of high-performance microporous polymer membranes. The large synthetic toolbox available for creating new polymers is simulated by translating the polymer into a binary “fingerprint,” which is input to the ML algorithm. The model is trained with a random subgroup of polymers from our literature database and then tested against the remaining polymers. The model is then applied to a large set of literature data to discover high-performance polymers, thus facilitating machine-assisted design. Reproduced without alternation from ref. [99] (CCL).

Barnett et al. [99], compiled a database of solubilities and permeabilities for 700 different polymers to several common gases (CH_4_, CO_2_, N_2_, O_2_, H_2_, and He). The polymers were composed of a series of standard building blocks (the selection of suitable monomers is not actually very wide). GPR was trained and tested using this database, which was randomly split into two groups in a standard manner. The model was then used to predict the permeability of 11,000 other polymers, which were randomly generated from the same building blocks (no systematic search was conducted). One of these candidate structures was synthesized, and it showed a high selectivity for CO_2_ over CH_4_, with a reasonable permeability above the Robeson bound (see Figure 5). Later, Yuan et al. [100], published a more extensive study using DTEs. 

**Figure 6:**
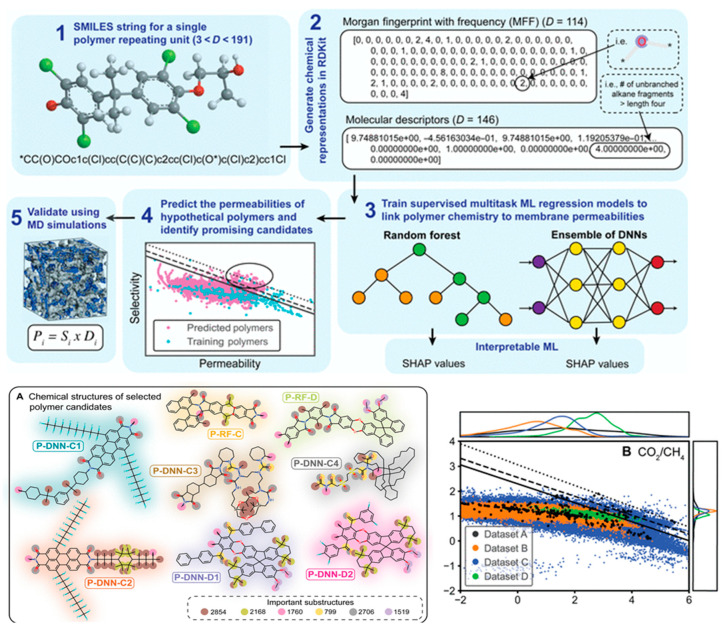
Workflow and the results of the search in chemical space for gas separation polymeric membranes. The top part demonstrated the workflow: SMILES are characterized by MFFs or RDKit descriptors [104] that serve as features. ML models are trained on the dataset of existing measurements and applied to the pools of available candidate polymers. The predictions are verified by MD simulations for selected candidates. Bottom left (**A**): candidate monomers selected with the ML algorithms. Bottom right (**B**): predicted selectivities for the training dataset and different pools of candidate structures (polyimides in blue, ladder polymers in green) with respect to the different version of the Robeson upper bound. Compiled from the figures of ref. [101] (CCL).

Yang et al. [101] attempted a search in the chemical space by modifying the SMILES string of the monomer and using a generative DNN. This approach proposed a wider pool of candidate structures. The authors used two methods to interpret the monomer’s structure: Morgan fingerprint with frequency (MFF) [102], which captures the frequency of chemical substructures present in molecules (114 features), and standard chemical descriptors for the polymer repeating unit, such as gyration radius, eccentricity, and asphericity (146 features). These “fingerprints” were used as features, and the permeabilities were the targets for training. The dataset contained 778 polymers with at least one measured gas permeability (335 unique monomers). The authors also compiled several datasets for which predictions were made. One of these was similar to the training set. Another consisted of 8+ million polyimides. The third was a set of 1,000+ ladder polymers [103]. RF and DNN models were trained on the training set and applied to the candite sets. The authors identified a number of candidate structures that were expected to exceed the Robeson tradeoff upper bound. They explored selected candidates using MD simulations, which for the most part agreed extremely well with the ML predictions. The workflow and selected results are shown in Figure 6.

Basgodan et al. [105] related the permeability to selectivity ratio to the glass transition temperature *T*_g_. using DTE-based approach. Very recent efforts by Jia et al. [106], Xu et al. [107], Cheung et al. [108], and Chen et al [109] feature generative DNN for the search in the chemical space of monomer structures and input from molecular modeling in the optimization algorithm. For example, in ref. [108] the molecular structures are obtained by energy minimization and then characterized using topological descriptors and rigidity. Molecular modeling links molecular structure of the polymer to the pore structure of the polymeric membrane material. It also makes the outcome more interpretable, which is important in molecular design. 

Very recently, Glass et al. [110] considered an effect of positively charges amine coatings on the performance of polymer membranes for water purification. This work is rather similar to the applications of ML to carbon optimization reviewed in Section 3. Chemical agents applied in membrane functionalization, their concentration and pKa were chosen as the independent features, while pure water permeation and zeta potential were the output characteristics. All experiments were performed in the same paper.

In summary, data-driven computational design of the microporous polymeric membranes has already contributed to shifting the Robeson bound upwards. It is also important to note that the polymers that were predicted to have superior selectivity and permeability have actually been synthesized, and the validity of these predictions has been proven (often, but not always [106]). Compared to carbons and other irregular nanoporous materials, the *in silico* data-driven design of microporous polymers enjoys a clear pathways for navigation. Although the experimental pool of available results is not large, a strong connection between the chemistry of the backbone and the performance metrics that are clearly established radically facilitate the application of data-driven methods. Molecular simulations aid in the characterization of monomers and the interpretation of results. An interesting future development could be a full-scale “molecular” design, where the shape of monomers is considered in a coarse-grained fashion using MD and related to pore structure. Databases of simulation results from coarse-grained models can easily be created, linking the structural elements of the polymer and the resulting porosity. Polymer chemistry can first be optimized *in silico* using crude models to obtain a reasonable PSD and throat distribution. Then, an atomistic representation can be employed to obtain shapes based on the results from the first stage. The complexity of the chemical space and the non-trivial relationship with performance make data-driven approaches essential for optimizing microporous polymer membranes.

## 5. ML in Synthesis and Optimization of Disordered Mesoporous Materials 

**Figure 7:**
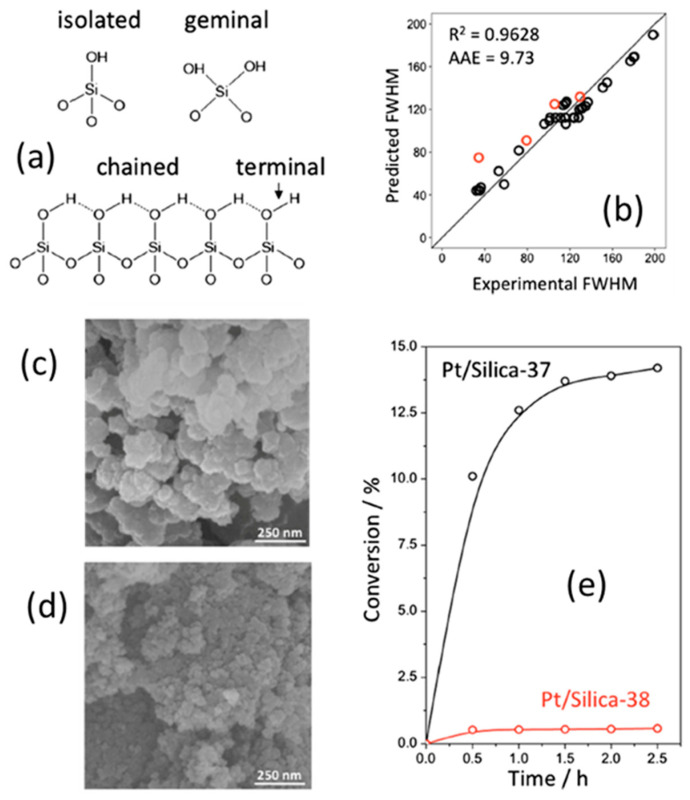
Illustration of ML application in the design of xerogel synthesis with maximized porosity and silanol group diversity, characterized by the full-width-half-maxima (FWHM) of infrared band. (**a**) silanol group types (**b**) SVM correlation between the characteristics of the original reactant formulation/synthesis procedure and the resulting FWHM. (**c**,**d**) SEM images of xerogels with maximized (Silica-37) and minimized (Silica-38) FWHM (**e**) catalytic performance of catalysts based on the samples shown in (**c**,**d**) panels illustration the importance of the silanol diversity. Despite a small dataset size, SVM correlations are instrumental in choosing the proper synthetic procedure, while multiple correlations based on two features are inconclusive. Compiled using images taken from ref [112] with a permission from Elsevier.

Mesoporous oxides have been well studied experimentally, and attempts to apply this knowledge to their design, screening, and optimization have been published in recent years. For instance, Sun et al. [111] prepared 77 samples of mesoporous alumina—silica ceramics using active carbon as a pore former, which was burned out after synthesis. The properties (mainly density) were then correlated with the initial mixture composition using several ML methods (k-nearest neighbors, KNN; support vector machine, SVM). Ten-fold cross-validation and random sampling techniques were employed to divide the dataset. While ML was introduced as a tool for porous ceramics design, it remains unclear how it actually helped. Wang et al. [112] synthesized 36 nanoporous silica xerogel samples using a segmented continuous flow reactor. The reactant mixture composition and synthesis process were adjusted to optimize yield and the concentration of silanol groups on the surface. The results were analyzed using SVM, which helped identify the reactant composition and synthesis conditions that maximize and minimize the diversity of the silanol group. The silanol group diversity influences the catalytic properties (see Figure 7).

Aerogels are highly porous materials synthesized by extracting the liquid component of a gel by means of supercritical drying or freeze-drying. This results in a continuous solid matrix (usually formed by a network of roughly spherical bodies) that occupies only a small fraction (0.1 to 10%) of the total material volume, with the rest being the pore space. The matrix materials widely vary, from inorganic oxides (SiO_2_, Al_2_O_3_, TiO_2_) to carbon. Due to their broad application range [113], ML-assisted design and synthesis of aerogels attracted substantial attention [114,115,116,117,118]. 

Younes et al [115] applied principle component analysis (PCA) to experimental results on ion removal from water with aerogels of different skeleton origin (cellulose, chitosan, graphene) and with different additives (such as MOFs). The database was limited to a dozen+ of samples explored experimentally [119], to which some other properties were added from the literature. The authors concluded the different samples were likely to perform approximately equally in ion removal with no feature (such as specific area or porosity) playing a critical role. PCA did not reveal much not seen by unaided eye, obviously because of the dataset size.

Tafreshi et al. [116] collected the details of the precursor (polymer, linker) chemistry and synthesis procedures for 60 different polyimide-based aerogel samples and attempted to relate the precursor chemistry and polymer concentration in the reactant mixture to the properties of the resulting material (such as density and porosity). They obtained very reasonable predictions using artificial neural networks (ANN). Considering a dataset of sixty samples and six easily controlled parameters, these good predictions are not surprising. However, the chemistry-structure relationship is important for future materials screening and optimization. Goodarzi et al. [118] collected chemical and structural data (aerogel density, solid matrix density, thermal conductivity, and pore scale) for 296 aerogels made from phenol formaldehyde, silica, polyvinyl chloride, resorcinol formaldehyde, polyurethane, and polyimides. They then used supervised machine learning methods to relate these parameters to the thermal conductivity of the aerogels. The results showed that the properties of the matrix material dominated the overall thermal conductivity. Gaussian process regression and nearest neighbor algorithms performed better than KNN. Han et al. [117] focused on the mechanical properties of concrete with aerogel additives. Due to the high uncertainty in the chemical composition of these materials, the authors considered both the precursor composition (e.g., water/binder ratio) and manufacturing procedures (aerogel and silica fume replacement rates) as features. A set of 660 data points was used for cross-validation and hyperparameter optimization through grid search. The water/binder ratio (as expected) was the most significant factor in determining the mechanical strength, while the aerogel replacement rate had the largest impact on thermal conductance. Ensemble learning, which is easier to interpret, performed better than traditional data analysis methods.

Rege et al. [120,121] applied purely computational approach to explore the structure–property relationships in aerogels. The aerogel formation was simulated as diffusion limited aggregation of spheres with sphere radius, concentration, seed step and walker step as the input parameters. DLCA lead to fractal structures characterized by fractal dimension and mechanical properties evaluated using finite-element modeling. The inverse problem of determining parameters that lead to the desired properties was solved via active learning. The fractal dimensions were substantially higher than typical to DLCA [122].

Finally, ML based optimization of aerogel applications also deserves mentioning. Zhou et al. designed an aerogel glazing system for thermal insulation in buildings [123]. Optimization was performed with a surrogate model based on supervised learning that replaced solution of thermal conduction PDEs and greatly accelerated the multivariant optimization. Uncertainties were estimated with Markov MC simulations. Later, the same authors introduced an ANN-based heuristic teaching-learning-based algorithm to optimize aerogel glazing systems using experimental observations from several different locations [124]. 

**Figure 8:**
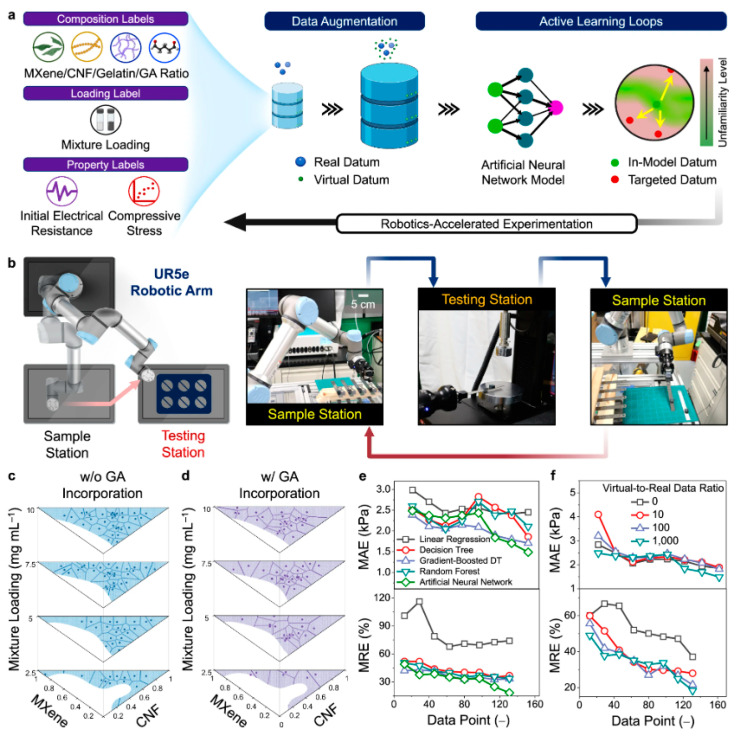
(**a**) Schematic illustration of a multi-stage ML framework for constructing a prediction model via active learning loops, data augmentation, and robot-human teaming. (**b**) An autonomous testing platform integrated with a robotic arm and a compression tester. 2D Voronoi tessellation diagrams (**c**) without and (**d**) with the glutaraldehyde incorporation after 8 active learning loops. (**e**) the mean absolute error (MAE) and the mean relative error (MRE) values of various prediction models based on linear regression, decision tree, XGBoost, RF, and ANN algorithms. (**f**) MAE (top) and MRE (bottom) values of various ANN models based on different virtual-to-real data ratios. Reproduced from ref. [125] (CCL).

Overall, the effort invested into mesoporous materials is relatively weak compared to the microporous ones. ML applications to mesoporous materials are still rare and far between, but worst of all, ML applications often lead to unclear outcomes (which we also found for carbons, Section 3). Practically everywhere, the training dataset is obtained in the same work and is of very limited size. The breakthrough may be envisioned in the area of database building (including structural properties like SAXS|SANS and adsorption isotherms at standard conditions) and the development of fast ML-assisted simulation tools, which is presumably easier for, say, aerogels than for irregular microporous solids. The other way to data-driven design improvement is robotized fabrication, very recently demonstrated by Shrestha et al. [125]. The authors reported an automated optimization of MXene [44,81,126]—aerogel composites powered by active learning (Figure 8). An automated pipetting robot was operated to prepare 264 mixtures of Ti_3_C_2_T_x_ flat sheet MXene, cellulose, gelatin, and glutaraldehyde at different loadings. After freeze-drying, the aerogels structural integrity was evaluated to train a support vector machine classifier. Using the samples as the initial leaning base, 162 more samples were synthesized in eight iterations, after each of which the new samples were added to the dataset. An ANN-based model for prediction of the resulting properties from the initial composition and synthesis conditions was constructed. In general, aerogel-based composites are attractive targets for ML based optimization [114,127] due to their complexity (compared to the traditional aerogels) and multitargeted applications (electric conductance in conjunction with thermal insulation). Active learning based robotized optimization is where data-driven approaches should excel, because a deterministic algorithm is very difficult to build and tune beforehand. Active research efforts should be expected in this area in the near future. 

## 6. Conclusions

**State of the art.** The paper reviews the current practices in ML application to irregular porous materials. It discusses the system types, goals, datasets, methods, achievements, and problems. The situation differs across different material classes. One important aspect is the lack of large established datasets that encompass the chemical composition, structural parameters, and properties of disordered porous materials or even separate classes of such materials. Some recent studies have started to develop seeds for future large datasets. With several exceptions, mostly dealing with microporous materials, ML methods are either (1) applied to small datasets of experimental data originating from the same work (2) applied to datasets obtained with simulations, with structures built with generative algorithms. Both groups contain a few good examples of data-driven design and optimization of porous materials. However, it is clear that at the moment, the data-based methods do not work up to their full potential.

**Path forward.** There are three obvious directions of improvement (i) database construction (ii) development of computational methods (iii) automated production and testing. Database construction is the primary direction for micro- and mesoporous materials like active carbons and mesoporous inorganic oxides. Widely available are X-ray/neutron scattering data, standard sorption isotherms, surface characteristics related to hydrophilicity (surface acidity for carbons [128]), sometimes adsorption selectivity for standard mixtures like CH_4_ + CO_2_, breakthrough curves and so on. Standard adsorption isotherms (N_2_ at 77 K, Ar at 87 K, CO_2_ at 273 K) allow PSD calculations. It is very important to have the original isotherms, from which PSD and SSA can be calculated in a reasonable and consistent manner. PSDs and SSA obtained in different papers are often inconsistent and sometimes obtained with methods not applicable to the particular material. The characterization data can be linked to the synthesis conditions and the performance (storage, separation, breakthrough, sound absorption, etc). 

Tools for cost estimation would significantly strengthen the industrial impact of ML in porous materials discovery and optimization. As always across the field, solutions should be different for different material types. The performance of carbons, microporous polymers for gas separation, and aerogels improves in small increments, and ML is hardly expected to change that. Massive production promises substantial economic gains even from small performance gains, and the cost of the new material plays an important role. Approximate cost evaluation should be embedded in the screening. The first steps towards cost estimation have been made already (e.g., ref. [101], which considers availability of synthesis, see Supplementary Info). The cost estimation is quite different from that for the computational drug design [129] purposes. Finally, automated production and testing of 3D structures is supposed to excel, especially in conjunction with active learning algorithms, and that has been demonstrated already [125]. Robotized synthesis is important both for database development and as the area of ML application to materials, since data-driven methods have become indispensable in optimization of complex processes with unclear interrelationships between the parameter.

**ML methods.** Only two ML tasks: (i) generation of new monomers during the search in chemical space of polymer backbones (ii) generation of model porous structures are computationally challenging and require the heavy machinery of deep DNN for disordered porous materials design and optimization. In most cases, the current datasets are too small for any advanced learning method to make a real difference. With some exceptions, as of today there is not too much to do for data science professionals. In a number of cases considered here, a multivariate regression of explicitly defined parametric form would probably work just as well. Even the best scenario of database development in the near future is unlikely to create truly challenging problems. Data-driven acceleration of computations with surrogate models, PIML or machine learned potentials in particle-based simulations [130] is another area, which is beyond the scope of this review. No matter how promising this direction is, most of the current efforts are still limited to demonstrative purposes and warrant some level of caution. At least for now, development of ML methodologies does not seem a direction of a potential breakthrough in data-driven design and optimization of disordered porous materials.

## Data Availability

No new data were created or analyzed in this study. Data sharing is not applicable to this article.

## Appendix A. Short Summary of Select Published Papers on Data-Driven Design and Optimization of Disordered Porous Materials

**Material**	**Porosity Type**	**Goal**	**Dataset (Source & Size)**	**ML Methods**	**Ref.**
Active carbons					
Granular active carbons	Toxic chemicals sorption in disordered microporosity, breakthrough	Relating early breakthrough time to easily measurable carbon characteristics air-hexadecane partition coefficient, hydrogen bond acidity	400 breakthrough curves for 18 carbons from literature, various pollutants	DTE	[86]
Granular active carbons	Disordered microporosity	Build correlation between source biomass, activation chemical, synthesis procedure and IAN	108 carbons, all sythesized in the authors lab	ANN, DTE	[86]
Activated carbons	Disordered microporosity	Build correlation between source biomass, synthesis procedure and yield + BET SSA	1500+ carbons from literature, all literature data	DTE	[65]
Activated carbons	H_2_ adsorption in disordered microporosity	Choosing best carbons for H_2_ adsorption, optimization of carbons	1700+ data points on H_2_ sorption on 68 carbons at 77 K and different *p*, features include composition, BET surface area, literature data	ANN	[77]
Activated carbons	H_2_ adsorption in disordered microporosity	Choosing best carbons for H2 adsorption, optimization of carbons	2072 data points on H_2_ sorption on 68 carbons at 77 K and different *p*, features include composition, BET surface area, literature data	DTE	[78]
Activated carbons	disordered micro- and meso-porosity	Examining the correlation between the electric double layer capacitance and the pore structure of active carbon	70 carbon samples from the literature; micro and meso pore SSA as features, capacitance as output at scan rates between 1 and 100 mV/s	DTE, ANN	[77,89]
Activated carbon and carbon molecular sieve	disordered micro- and meso-porosity	Building a surrogate model to predict sorption isotherms	1200+ measurements of N_2_, O_2_ and N_2_O adsorption	ANN	[85]
Carbide-derived active carbonds	disordered micro- and meso-porosity	Correlation ciprofloxacin sorption capacity to strutural parameters (SSA, average pore size, total pore volume, micropore volme, called “texture”, their origin unfortunately unclear)	87 different carbon samples, ciprofloxacin adsorption capacity	Linear model	[131]
Activated carbons	disordered micro- and meso-porosity	Examining the correlation between the electric double layer capacitance and the pore structure of active carbon	70 carbon samples from the literature; micro and meso pore SSA as features, capacitance as output at scan rates between 1 and 100 mV/s	DTE, ANN	[77,89]
Mesoporous materials					
Porous Al_2_O_3_/SiC ceramic cakes	Irregular nanoporosity, honeycomb-like structure	Linking synthesis conditions to porosity and Al_2_O_3_ content	50 samples, all experimentally obtained in the same work	ANN	[132]
Nanostructured hydrogel	Interstitial (semi-regular, formed via amphiphilic segregation)	Optimization of printing parameters	Experiment, from the same work, 12 points	SVM	[133]
polyimide based aerogels	Disordered mesoporosity, highly porous medium	Optimization of reactant formulation and synthesis conditions to obtain best performing material	60 different hydrogels, all measured in the same paper	ANN	[116]
SiO_2_-Al_2_O_3_ porous ceramics	Irregular mesoporosity	Optimization of reactant formulation and synthesis conditions to obtain best performing material	77 samples, all from the same study	Diffe-rent me-thods	[111]
Simulated aerogels		Far goal—optimize pore structure; near—predict Df on simulations parameters	3125 simulated aggregates of spherical particles, 3 features	ANN	[120]
polyurethane aerogel, silica–resorcinol formalde-hyde aerogel	Irregular mesoporosity	Link gel density, solid phase density & conductivity to everall thermal conductivity	Several dozen, all synthesiszed in the same study	KNN, GPR	[118]
silica xerogels	Disordered mesoporosity	Optimize reactants compositions, synthesis procedure was adjusted to optimize the yield and silanol group surface concentration	36 synthesized samples. All from the same work	SVM	[112]
Cellulose, chitosan and graphene based aerogels with microporous additives	Disordered mesoporosity, in some samples ordered microporosity	Evaluation of different factors influencing; Aerogels Efficiency towards Ion Removal	17 samples, from literature	PCA	[115]
Aerogel incorporated concrete	Disordered mesoporosity, disordered macroporosity	Elucidating factors determining thermal conductivity, mechanical properties	660 samples, experimentally studied	DTE	[117]

## Appendix B. Lay Person Overview of the Main Machine Learning Methods Applied in Data-Driven Design, Characterization and Optimization of Disordered Porous Materials

### Appendix B.1. Supervised Learning Methods

ML in the reviewed papers is solely used to build regression models, linking structural parameters to properties, and parameters characterizing the synthesis/production process to structure and processes. The following methods are most popular:

Support Vector Machine [134] (SVM). Uses kernel transformation of the data to multi-dimensional feature space where effective linear approximation is possible. A very common classical method for regression problems resilient to noisy data.

*k* nearest neighbors (KNN): the output value is the weighted or unweighted average of the values of *k* nearest neighbor of that location. *k* is a regularization parameter chosen to minimize the prediction error for the test set.

Decision tree ensembles (DTE): DT regressor creates a tree-like structure of decisions based on input features, where each internal node represents an attribute, and each leaf node contains a prediction. The algorithm recursively partitions the data into smaller subsets until reaching a stopping criterion. Tree depth is the main regularization parameter chosen to maximize the prediction accuracy. Most common DTE methods are random forest (RF) [135], which combines output of several random decision trees into a single result) and gradient boosting, [136] where next decision tree generation minimizes the error obtained by the previous tree generation.

Gaussian process regression (GPR, aka kriging): a method of interpolation based on Gaussian process governed by prior covariances. Under suitable assumptions of the prior, kriging gives the best linear unbiased prediction (BLUP) at unsampled locations.

Artificial neural network (ANN) consists of layers of artificial neurons connected by edges. Each artificial neuron receives signals from the connected neurons, then processes them and sends a response signal (in a form of a real number multiplied by an optimizable weights). The signals from the output layer is the outcome, the weights are optimized to minimize the error. The ANN topology (number and the size of layers and their connectivity) and the activation function (define the signal processing by the particular neuron) is chosen for the particular problem. ANN hold advantage over other methods on large datasets with many features, but are most expensive to train. A generative adversarial network (GAN) [137] two neural networks contest with each other in the form of a zero-sum game: one is the generator (generates structures close to the original distribution) and the discriminator network evaluates them. The discriminator is trained on the samples from the target distribution and evaluates the likelihood that the particular sample belongs to that distribution, while, the generator is trained to produce the samples that discriminator cannot distinguish (that is, gives high likelihood estimates).

Active learning (AL): [138] the algorithm queries the subject of learning to obtains the output for a one or several inputs; the responses are added to the dataset, the model is adjusted and a new query submitted until convergence criteria are satisfied. Most commonly based on Bayesian optimization, which treats a black box system as a random function described with a probability distribution that captures beliefs on the system behavior. After receiving responses to the queries, the distribution is updated and used to construct an acquisition function to formulate the next query.

### Appendix B.2. Unsupervised Learning Methods

Principal component analysis (PCA) [139,140] is a linear dimensionality reduction technique, where the data is linearly transformed onto a new coordinate system in such a way that the directions (principal components) capturing the largest variation in the data can be easily identified.
